# The relationships between growth rate and mitochondrial metabolism varies over time

**DOI:** 10.1038/s41598-022-20428-9

**Published:** 2022-09-27

**Authors:** Jean-Baptiste Quéméneur, Morgane Danion, Joëlle Cabon, Sophie Collet, José-Luis Zambonino-Infante, Karine Salin

**Affiliations:** 1 Ifremer, Laboratory of Environmental Marine Sciences, University Brest, CNRS, IRD, 29280 Plouzané, France; 2 Anses, Ploufragan-Plouzané Niort Laboratory, VIMEP Unit, Technopôle Brest-Iroise, 29280 Plouzané, France

**Keywords:** Energy metabolism, Ecophysiology, Physiology, Zoology

## Abstract

Mitochondrial metabolism varies significantly between individuals of the same species and can influence animal performance, such as growth. However, growth rate is usually determined before the mitochondrial assay. The hypothesis that natural variation in mitochondrial metabolic traits is linked to differences in both previous and upcoming growth remains untested. Using biopsies to collect tissue in a non-lethal manner, we tested this hypothesis in a fish model (*Dicentrarchus labrax*) by monitoring individual growth rate*,* measuring mitochondrial metabolic traits in the red muscle, and monitoring the growth of the same individuals after the mitochondrial assay. Individual variation in growth rate was consistent before and after the mitochondrial assay; however, the mitochondrial traits that explained growth variation differed between the growth rates determined before and after the mitochondrial assay. While past growth was correlated with the activity of the cytochrome c oxidase, a measure of mitochondrial density, future growth was linked to mitochondrial proton leak respiration. This is the first report of temporal shift in the relationship between growth rate and mitochondrial metabolic traits, suggesting an among-individual variation in temporal changes in mitochondrial traits. Our results emphasize the need to evaluate whether mitochondrial metabolic traits of individuals can change over time.

## Introduction

Growth performance is closely related to an individual's fitness by influencing body size and, in turn, age at maturity, fecundity rate, foraging ability, and predator escape^1,2^. The physiological processes involved in somatic growth such as cell division and protein synthesis require ATP, which is mainly produced by the mitochondria^3^. Interestingly, individuals that are most efficient at converting nutrients into ATP will typically have faster growth than those that do so less efficiently^4,5^. This can occur either directly via higher mitochondrial efficiency of ATP production (experimentally tracked as the amount of ATP generated per molecule of oxygen consumed, ATP/O ratio) or indirectly through higher rates of oxygen consumption (OXPHOS) for ATP production or more mitochondria^6,7^. Conversely, variation in the availability in ATP between individuals might also occur because individuals differ in the proportion of the oxygen that is consumed by mitochondria to offset the proton leakage (LEAK respiration) across the inner mitochondrial membrane^8^. In each case, the somatic growth can be upregulated from more cell division and protein synthesis and hence biomass production with availability in ATP.

Our current understanding of the relationship between mitochondrial properties and growth performance is largely based on studies of growth trials that occur over a period of time anterior to mitochondrial measurement^4,5,9^, hereafter called past growth. The main reason for this caveat is that studying mitochondrial metabolic traits usually involves terminal sampling. If mitochondrial properties are only determined toward the end of the growth trial, it is the variation in growth performance that might actually promote differences in mitochondrial metabolic traits among individuals. Hence, ascertaining the causality in a relationship between mitochondrial metabolic traits and growth performance in the absence of a clear temporal relationship can be a source of debate. Previous work in tadpoles has demonstrated that experimental variation in mitochondrial metabolic traits lead to variation in future growth^10^. However, it is also likely that mitochondrial variation induced by an artificial substance, the 2,4-Dinitrophenol, is not representative of natural variation in mitochondrial metabolic traits. Natural variation in mitochondrial metabolic traits might be related to individual variation in future growth, and, in turn, predict the rate of growth subsequent to the mitochondrial assay, but that has not been documented previously.

Investigations of the relationship between mitochondrial metabolic traits and growth performance have generally focused on a growth trial that represents a relatively short period of time. For example, individual brown trout and broilers that had lower rates of LEAK respiration exhibited better growth performance, where growth was measured over 11 to 20 days in trout^11^ and 7 days in broilers^5^ before the measurement of mitochondrial function. Similarly, study in juvenile brown trout demonstrates a positive association between mitochondrial ATP/O ratio and growth, but growth was determined over a trial of 14 days for a trout that can live for years^4^. Growth rate is generally repeatable over time when measured under constant conditions^12^. However, metabolic traits of mitochondria are flexible and can change dramatically in as little as few hours^13^. Experimental studies now show that mitochondrial metabolic traits can change in less than a month in different taxa, including insects^14^, bivalves^15^, fishes^16^, birds^17^ and mammals^18^. However, individuals can differ in the degree to which their mitochondrial traits change as a function of time and therefore the link between mitochondrial traits and growth performance may decline over time.

In this study, we measured mitochondrial metabolic traits using a non-terminal technique for tissue sampling in fish, which can then be used to measure performance following the sampling time. Using European sea bass (*Dicentrarchus labrax*), we studied whether individual variations in past and future growth rates were related to individual variations in mitochondrial metabolic traits. We tested the predictions that (i) individual variation in growth rate, both before and after the mitochondrial assay, is related to individual variation in similar mitochondrial traits, and (ii) the relationship between growth rate and mitochondrial traits declines over time. To test these hypotheses, we examined the growth rates up to 20 weeks before and 12 weeks after mitochondrial measurement, respectively. We analyzed mitochondrial metabolic traits (rates of OXPHOS and LEAK respiration, ATP production, and cytochrome c oxidase (COX) activity, a measure of mitochondrial density) in the red muscle. We chose this tissue because it can be sampled using biopsy punches to collect a muscle tissue plug in a non-lethal manner^19,20^ and has, in contrast to white muscle, a particularly high mass-specific mitochondrial metabolism^21,22^.

## Methods

The experiments were approved by the French Ethics Committee in charge of Animal Experimentation (no.2019072411491441) and were in accordance with institutional and ARRIVE guidelines.

### Animal collection and husbandry

In May 2019, juvenile European sea bass, *Dicentrarchus labrax* (Linnaeus 1758) (6 months old, mass 5 g), were transferred from a fish farm (Turbot Ichtus, Trédarzec, France) to the Ifremer rearing facility (Plouzané, France). Fish were kept in a common tank for 5 months, maintained under a 12 L: 12 D photoperiod, and fed at satiety three times a week using commercial pellets (Neo Start, Le Gouessant, Lamballe, France).

In October 2019, fish (n = 40) were anaesthetized (Tricaïne; 125 mg L^−1^), weighed (41.5 ± 1.8 g, MCE11201S-2S00-0, Sartorius, Göttingen, Germany), and implanted subcutaneously with an identification tag (RFID; Biolog-id, Bernay, France). The fish were then randomly allocated to ten replicate 400 L tanks supplied with open-flow, fully aerated seawater (oxygen saturation > 95%, salinity 32 ppt), thermo-regulated during winter to avoid falling below 13 °C, and fed at satiety three times a week. Temperature was recorded weekly. To account for the potential effect of temperature variation over the duration of the trial (15.5 ± 0.5 °C, range: 13.1–17.9 °C) on growth, a correlations analysis was performed between temperature and specific growth rate (SGR). No statistical relationship was found between SGR and temperature (Spearman *R*^*2*^ = 0.060, *P* = 0.596). Additional fish (n = 40) were present in the tanks (final density: n = 8 per tank) for the need of another project.

### Growth measurements

Body mass (BM) was measured about every four weeks from October 2019 to June 2020. The fish were fasted for 48 h and anesthetized before each BM measurement (± 0.1 g). The specific growth rate (% day^-1^) was estimated as follows:

$${\text{Specific~Growth~Rate}} = ~\frac{{\ln \left( {final~BM} \right) - \ln \left( {initial~BM} \right)}}{{{\text{days~elapsed}}}} \times 100$$

**Figure 1:**
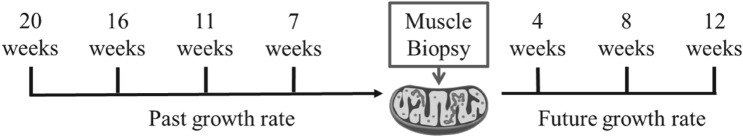
Experimental design. Juvenile European sea bass (n = 40) were weighted about every four weeks over a 32-week period. At week 20, a biopsy of red muscle was used for mitochondrial assay. Specific growth rates (SGR) were calculated relative to the time of the biopsy. Past growth rate corresponds to SGR calculated before the biopsy, and future growth rate corresponds to SGR calculated after the biopsy.

In March 2020, a red muscle biopsy sample was collected from fish to measure the mitochondrial metabolic traits. Past growth was defined as specific growth rates before the analysis of mitochondrial metabolic traits (past specific growth rate, SGR_past_). SGR_past_ were calculated using the BM at the muscle biopsy as the final BM and the BM at 7, 11, 16, and 20 weeks before the muscle biopsy as the initial BM (Fig. 1). Future growth was defined as specific growth rates after analysis of mitochondrial metabolic traits (future specific growth rates, SGR_future_). SGR_future_ were calculated using the BM at 4, 8, and 12 weeks after the muscle biopsy as the final BM and the BM at the muscle biopsy as the initial BM. In European sea bass, most of the somatic growth occur within the first 3 to 5 years of life, so several months of growth measurement at the juvenile stage might be representative of the overall growth of the animal.

### Muscle biopsy procedure

Muscle biopsy was performed as a non-lethal means of sampling tissue for the mitochondrial assay while allowing us to determine future growth rate. Fish were anaesthetized with tricaine (as above), weighed (76.7 ± 3.6 g), and biopsied. A skin incision (< 10 mm in length) was made with a scalpel below the lateral line and between the dorsal and caudal fins. Then, a core of the red muscle was collected using a biopsy punch (2 mm LCH-PUK-20, Kai Medical, Solingen, Germany). The core of red muscle was immediately cleaned of white muscle with a scalpel. The red muscle sample was then weighed (5.6 ± 0.2 mg; AC210P-0F1, Sartorius, Göttingen, Germany), and transferred to ice-cold respiration buffer (20 mmol L^−1^ Taurine, 10 mmol L^−1^ KH_2_PO_4_, 20 mmol L^−1^ HEPES, 110 mmol L^−1^ D-sucrose, 60 mmol L^−1^ K-lactobionate, 1 g L^−1^ BSA fatty acid free, and pH 7.0 at 13 °C). The incision was disinfected (Vétédine®Solution, Vetoquinol, Magny-Vernois, France) and filled with powdered bandage (ORAHESIVE, ConvaTec®, Deeside, UK). The fish were placed in a recovery tank before returning to their original tank.

The newly developed sampling procedure for mitochondrial assay required several preliminary works. A pilot experiment on a different set of fish evaluated the immunological consequences of biopsy on inflammation resulting from bacterial infection or damaged tissue. There was no significant effect of the biopsy on the immune system (see the supplementary materials and methods for details of all assay protocols and results Fig. S1b). This experiment also demonstrated that biopsy slightly reduced the growth rate but fish continued to gain mass (Fig. S1a). Finally, fish needed anesthesia for the biopsy procedure, and tricaine is the most judicious to study mitochondria in fish muscle. Tricaine acts by blocking voltage-gated sodium channels and blocks neural action potentials^23^. Muscle sodium channels are relatively insensitive to tricaine^23^, which suggest that tricaine are unlikely to affect the physiology of muscle cells. However, it would be relevant to test for potential effect of tricaine on mitochondrial function in fish muscle.

### Tissue homogenate for mitochondrial assay

Immediately after biopsy, muscle samples were shredded using micro-dissecting scissors to obtain a homogenous solution with a particle size less than 0.5 mm (tested by pipetting through a 1 mL tip), an homogenization procedure adapted from^24^. The tissue preparation by shredding used here allowed a rapid and efficient muscle permeabilization, with no loss of tissue, while maintaining the quality of the mitochondria. Validations of the permeabilization method are described in^11,25^. The homogenates were diluted further in respirometry buffer to obtain a final concentration of 1 mg mL^−1^ (mean ± s.e = 1.0 ± 0.2 mg mL^−1^). Red muscle homogenizations were carried out on ice. Only four fish could be run simultaneously for the mitochondrial assay (one measurement per fish), and two runs per day were performed; therefore, five days were required to analyze the forty fish.

### Measurement of mitochondrial metabolic traits

Oxygen consumption and magnesium green fluorescence were detected simultaneously in four respirometry chambers (2 mL, a chamber per fish) equipped with fluorescent sensors and recorded using DatLab software (Oroboros Instruments, Innsbruck, Austria) at 13 °C with continuous stirring. Immediately after homogenisation and dilution, 2.2 mL of the homogenate was added to one of the four chambers. Mitochondrial metabolic traits were determined using the method described by *Chinopoulos, *et al.^26^. First, an adenylate kinase inhibitor (P1,P5-Di(adenosine-5′) pentaphosphate pentasodium salt, 0.1 mmol L^−1^), complex I-linked substrates (pyruvate 5 mmol L^−1^ and malate 0.5 mmol L^−1^), and MgGreen (2.2 µmol L^−1^) were added to each well. After addition of EGTA (0.1 mmol L^−1^) and EDTA (15 µmol L^−1^), the fluorescent signal of MgGreen was calibrated using ten successive injections of MgCl_2_ (1 mmol L^−1^). Succinate (10 mmol L^−1^) was added to supply complex II with the energy substrates. OXPHOS respiration and ATP production were measured by adding ADP (2 mmol L^−1^). Leak respiration was measured by inhibiting ATP synthesis by adding carboxyatractyloside (4 µmol L^−1^). The rate of ATP disappearance owing to ATPase activity was also measured under these conditions. This measurement was subtracted from ATP production measured during OXPHOS respiration. An inhibitor of coenzyme Q-cytochrome c reductase (CIII), antimycin A (2.5 µmol L^−1^), was then added. This measure was subtracted from other mitochondrial respiratory rates to correct for oxygen consumption unrelated to the respiratory chain activity. Finally, mitochondrial density was estimated with COX activity by adding ascorbate (8 mmol L^−1^) followed by N,N,N,N-tetramethyl-p-phenylenediamine (TMPD; 0.5 mmol L^−1^) to the chambers. The rate of ATP production and ATP/O ratio were calculated as described in Salin, et al.^27^. Briefly, we converted the fluorescent signal of the free magnesium concentration to the ATP concentration. We used the same binding affinity (K_d_) for Mg^2+^ bound to ATP and ADP as in Thoral, et al.^28^ because the mitochondrial analyses were performed under the same conditions of temperature and homogenate concentration (K_d-ATP_ = 0.266 mmol L^−1^, K_d-ADP_ = 1.803 mmol L^−1^). The ATP/O ratio was calculated as the ratio of ATP production to OXPHOS respiration. Rates of oxygen consumption and ATP production were expressed as pmol s^− 1^ mg^− 1^ wet tissue.

In an additional experiment, we determined the technical repeatability of mitochondrial metabolic traits. Duplicated measurements of mitochondrial metabolic traits of two biopsies from the same individual were significantly reproducible (Intraclass correlation coefficients: OXPHOS respiration *r* = 0.41, *p* < *0.001*; LEAK respiration *r* = 0.48, *p* < *0.001*; COX activity: *r* = 0.40, *p* < *0.001*; n = 19; ATP production *r* = 0.44, *p* = *0.001*; n = 14).

### Statistical analysis

Intra-class correlation (ICC) was used to test the consistent differences in growth rates among individuals before and after the mitochondrial assay. Individual consistency of growth rate was tested between the most similar duration of the growth trial, that is, SGR_past_ at 7 and 11 weeks was correlated to SGR_future_ at 8 and 12 weeks, respectively. Linear mixed models (LMMs) were used to determine the relationships between fish growth rate and mitochondrial metabolic traits. Each SGR (SGR_past_ over 7, 11, 16, and 20 weeks, and SGR_future_ over 4, 8, and 12 weeks) was run in separate models. The models included SGR as the dependent variable, OXPHOS respiration, ATP production, LEAK respiration, and COX activity as continuous predictors, with the fish tank as a random factor. To control for the effect of initial body mass on growth rate, initial body mass was included as a covariate in each model. The day and the processing run of the mitochondrial assay, as well as the Oroboros device, and the respirometry chamber used for the analysis of mitochondrial assay were initially included as potential random factors in each model but were not significant and were subsequently removed. We also tested whether the degree of mitochondrial efficiency in generating ATP, as determined by the ATP/O ratio, explained individual variation in growth, which had to be examined in a separate linear model to prevent problems associated with multicollinearity between ATP/O, OXPHOS respiration, and ATP production.

Since mitochondrial density can influence mass-specific rates of mitochondrial metabolic traits (Pearson correlations: all r > 0.37 and *p* < 0.05) and SGR. Estimate of COX-independent mitochondrial traits were used in preliminary LMM analyses of SGR. Residuals from separate linear regressions relating mitochondrial metabolic traits (e.g. OXPHOS and LEAK respiration and ATP production) to COX activity were calculated. As before, we used LMM with SGR as the dependent variable and COX-independent OXPHOS and LEAK respiration, COX-independent ATP production, and COX activity as continuous predictors. Since the patterns of the results of growth rate analyses were the same whether mitochondrial metabolic traits were measured in terms of mass-specific or COX-independent tissues, only those for mass-specific mitochondrial metabolic traits are reported here.

All analyses were based on a sample size of 40 fish and all statistical analyses were performed using R (4.0.3; package lme4; package partR2). Data are presented as means ± SEM, the significance level was set to *p* < 0.05, and inclusive R^2^ (IR^2^) was added to illustrate the variance explained by a predictor in a model^29^.

## Results

**Figure 2:**
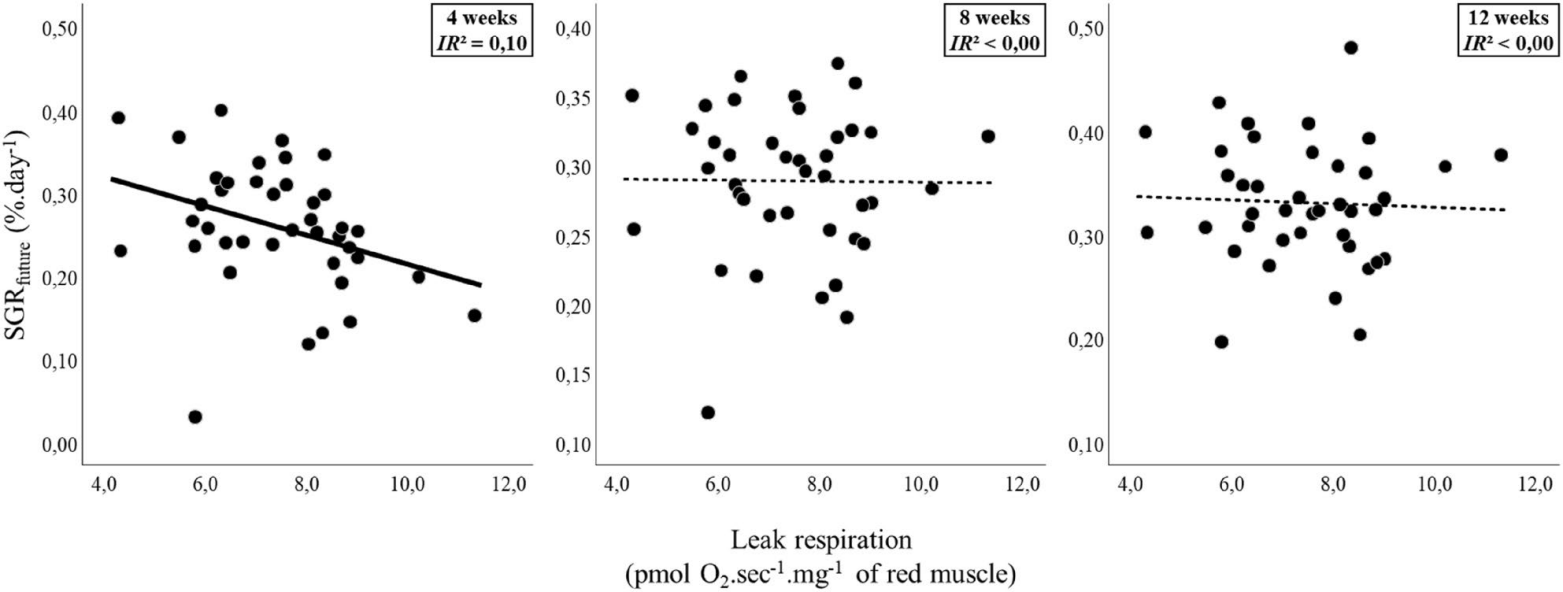
Future growth rates over 4 weeks was negatively related to the mitochondrial LEAK respiration of red muscle. Future Specific Growth Rates (SGR_future_) as a function of variation in Leak respiration in red muscle of juvenile European seabass (n = 40). SGR_future_ were calculated between body mass measured in the mitochondrial assay and posterior body masses. Continuous lines show significant effect (*p* < 0.05) and inclusive R^2^ (IR^2^) were added to illustrate the variance explained by LEAK respiration in the variation of SGR_future_. See Table S1 for statistical analyses.

Individuals varied considerably in their past and future growth rates (Table 1), but as expected, individual variation in growth was significantly repeatable before and after the mitochondrial assay (ICC: all *r* > 0.31 and *p* < 0.05). The consistency in the rank order of an individual growth rate between before and after the biopsy procedure shows that the biopsy procedure did not impact the inter-individual differences in growth. Future growth rate was negatively correlated with LEAK respiration (Fig. 2, Table S1). Indeed, variations in LEAK respiration predicted the SGR_future_ at 4 weeks (t_34.00_ = −2.23, *p* = 0.033, IR^2^ = 0.10; Table S1). The relationship between SGR_future_ and LEAK respiration was no longer significant after 8 and 12 weeks of growth (Table S1). Future growth rates were unrelated to OXPHOS respiration, ATP production, or COX activity in the muscle mitochondria (Table S1).

**Table 1:** Descriptive analyses of specific growth rates (SGR) and mitochondrial metabolic traits (OXPHOS respiration, ATP production, ATP/O ratio, LEAK respiration, and cytochrome c oxidase [COX] activity) in red muscle of juvenile European sea bass (n = 40).

	N	Min	Max	Mean ± SE
**Past growth rates**				
SGR_past_ 20 weeks (% day^−1^)	40	0.21	0.30	0.37 ± 0.08
SGR_past_ 16 weeks (% day^−1^)	40	0.22	0.49	0.36 ± 0.07
SGR_past_ 11 weeks (% day^−1^)	40	0.21	0.54	0.34 ± 0.07
SGR_past_ 7 weeks (% day^−1^)	40	0.12	0.42	0.30 ± 0.07
**Future growth rates**				
SGR_future_ 4 weeks (% day^−1^)	40	0.03	0.40	0.26 ± 0.08
SGR_future_ 8 weeks (% day^−1^)	40	0.12	0.37	0.29 ± 0.05
SGR_future_ 12 weeks (% day^−1^)	40	0.20	0.48	0.33 ± 0.06
**Mitochondrial metabolic traits**				
OXPHOS respiration (pmol O_2_ s ^−1^ mg^−1^ red muscle)	40	37.16	98.37	67.21 ± 15.03
ATP production (pmol ATP s ^−1^ mg^−1^ red muscle)	40	65.02	387.67	176.60 ± 63.20
ATP/O ratio	40	0.53	2.44	1.28 ± 0.33
LEAK respiration (pmol O_2_ s ^−1^ mg^−1^ red muscle)	40	4.27	11.30	7.40 ± 1.48
COX activity (pmol O_2_ s ^−1^ mg^−1^ red muscle)	40	72.53	118.01	92.13 ± 10.46

**Figure 3:**
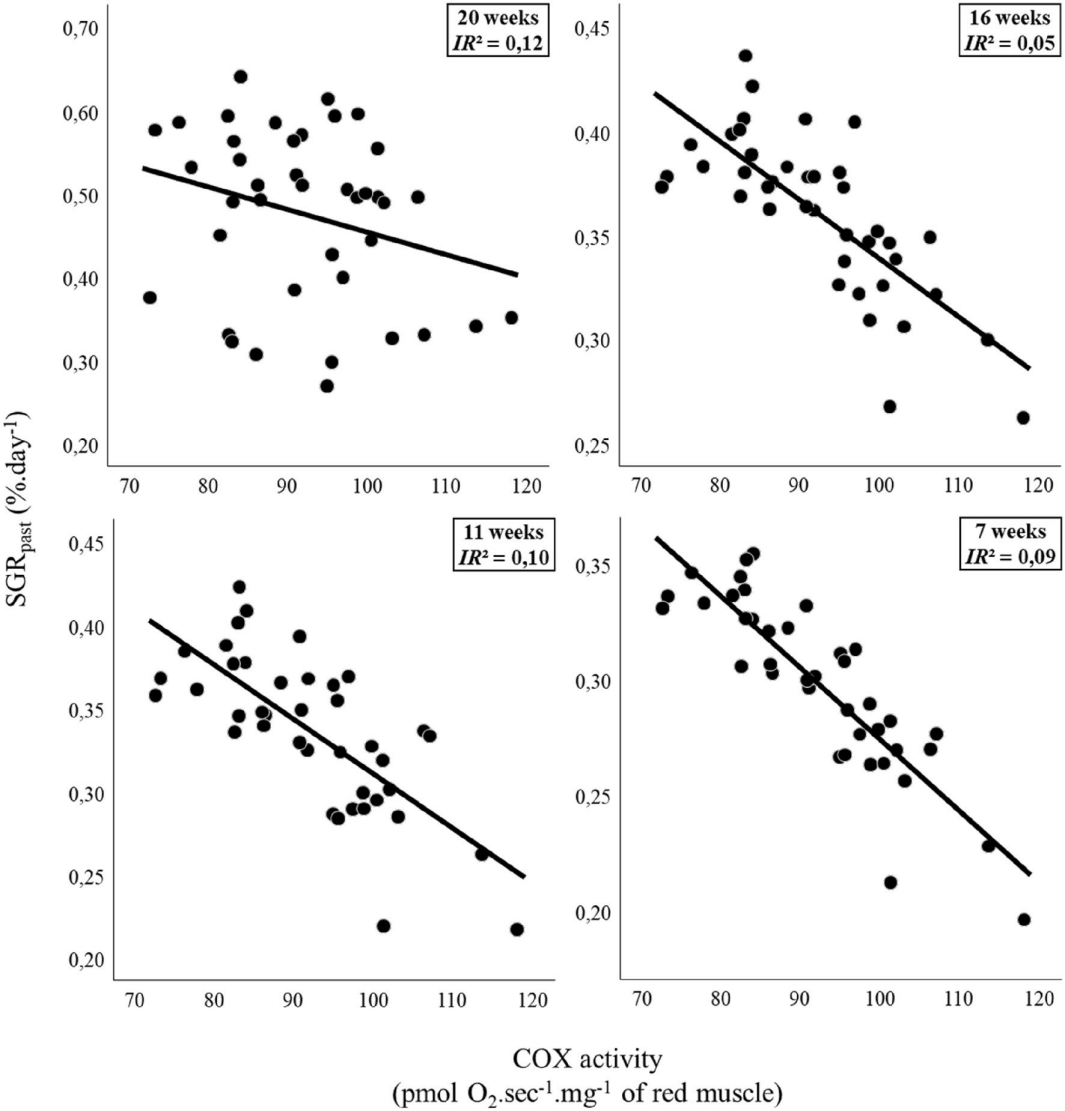
Past growth rates were negatively related to cytochrome c oxydase activity of red muscle mitochondria. Past Specific Growth Rates (SGR_past_) as a function of variation in Cytochrome C Oxidase (COX) activity in red muscle of juvenile European seabass (n = 40). SGR_past_ were calculated between body mass measured before the mitochondrial assay (at 20, 16, 11, and 7 weeks before) and the body mass at the mitochondrial assay. Duration of the growth trials is indicated on the top right of each panel. For 20 weeks period, plotted represent SGR_past_ since initial body mass has not significant effect on SGR. For 16, 11 and 7 week periods, SGR is plotted as partial residuals of SGR_past_ evaluated at mean initial body mass (46.6 g, 53.8 g, 60.8 g, respectively) since initial body masses had significant effect on SGR. Continuous lines show significant effect (*p* < 0.05) and inclusive R^2^ (*IR*^*2*^) were added to illustrate the part of past SGR variation explained by COX activity. See Table S2 for statistical analyses.

Individuals with the fastest past growth had lower COX activity in their red muscle mitochondria (Fig. 3, Table S2). Indeed, variations in SGR_past_ at 7, 11, 16, and 20 weeks were negatively correlated with COX activity (7 weeks: t_31.07_ = −3.68, *p* < 0.001, IR^2^ = 0.09; 11 weeks: t_31.96_ = –−3.98, *p* < 0.001, IR^2^ = 0.10; 16 weeks: t_33.82_ = −3.46, *p* = 0.001, IR^2^ = 0.05; 20 weeks: t_33.58_ = −3.79, *p* < 0.001, IR^2^ = 0.12; Table S2). Past growth rates were unrelated to OXPHOS respiration, LEAK respiration, or ATP production in the muscle mitochondria (Table S2).

## Discussion

We accepted our hypothesis that individual variations in both past and future growth rates are related to mitochondrial metabolic traits. However, the mitochondrial traits that explained the growth variation differed between the growth rates determined before and after the mitochondrial assay. While past SGR was correlated with COX activity, future SGR was linked to LEAK respiration in the red muscle mitochondria. Nonetheless, as predicted, individuals with the fastest future growth had lower LEAK respiration rates than fish that grew slowly after the mitochondrial assay. However, contrary to expectations, individuals with the fastest past growth had lower COX activity than fish that grew slowly before the mitochondrial assay. We partially accept our hypothesis that the relationship between growth rate and mitochondrial properties declines over time, with the decline being especially marked for future growth rates (after four weeks), while that relationship persists over 20 weeks of past growth rates.

Low LEAK respiration may confer an advantage for future growth. Our fish showed a negative relationship between the rates of LEAK respiration and future growth, calculated as the gain in mass between the time of the mitochondrial assay and four weeks later. This suggests that individuals with higher proton leakage dissipate more energy to offset leakage in place of growth, as previously shown in fish^11^, birds^9^ and mammals^30^. But, we also found that this relationship did not persist after eight weeks of growth. The mitochondrial metabolic traits of an individual can change over time^17,31,32^. However, plastic changes are often not similar across individuals^33,34^. Individuals might differ in mitochondrial plasticity simply as a result of changes in mitochondrial traits over time as animals grow or interact in their social environment.

Individual variations in past growth periods were not a function of mitochondrial LEAK respiration. Rather, past SGRs were negatively related to COX activity. This negative relationship is inconsistent with previous findings that individuals with the fastest growth have higher COX activities than fish that grow slowly^6,7,35^. Although the relationships between growth and metabolic traits have generally been studied in the mitochondria of white muscle^6,35^, liver and intestine^35^ , and the whole body^36^, one would expect the rank order of an individual’s red and white muscle mitochondria traits, (*i.e.* its mitochondrial metabolic traits in red and white muscle relative to other individuals in the population) to be consistent between tissues. However, there is also increasing evidence of the absence of covariation in mitochondrial metabolic traits between tissues of the same individual^11,25^ as well as evidence of tissue-specific relationships between mitochondrial metabolic traits and whole-animal performance^4,25^. Since our data are correlative, we cannot rule out that intrinsic differences in growth may drive variations in tissue composition if some individuals have more fat reserves and, therefore, lower mass-specific mitochondrial density. An alternative explanation might lie in the fact that activity of the COX is representative of rates of substrate oxidation, where correlation of markers of mitochondrial density can be poor^37^ (but see^38^). While measurements of several markers of mitochondrial density were beyond the scope of the present study, citrate synthase activity can be performed from the homogenate after the respirometry protocol. In this way, mitochondrial respiration and CS activity can be determined on the same tissue preparation. Clearly, more research is needed to ascertain whether individual variations in growth are expected to have important consequences for mitochondrial density in some tissues.

Our study questions the idea that mitochondrial metabolism is the underlying mechanism of individual variation in growth measured before the mitochondrial assay. The differential effect of mitochondrial metabolic traits on past and future growth is a complex but highly relevant issue, since almost all studies that have examined the effect of mitochondrial metabolism on animal performance have determined mitochondrial metabolic traits after, and not before, animal performance. These differential effects occurred despite our experimental animals being of similar age and nutritional state, and having been maintained under identical environmental conditions before and after mitochondrial analyses. Temporal changes in mitochondrial phenotype might occur within weeks^39^, days^17^ and hours^31^. Since the fish growth were determined over 5 months, we cannot rule out that internal clocks of the sea bass, despite relatively constant rearing conditions, played a role in influencing mitochondrial metabolic traits. Little is known about the temporal variation of the mitochondrial phenotype, but Stier, et al.^17^ found repeatability in mitochondrial metabolic traits over a period of 10 days in the red blood cells of pied flycatchers (*Ficedula hypoleuca*). Knowledge regarding how mitochondrial traits change over time is currently a crucial step for future research on the relevance of mitochondrial metabolic traits for organismal performance.

Our data emphasize the importance of information regarding the temporal repeatability and variation of mitochondrial metabolic traits. Such data are still lacking because of the need to cull animals when sampling tissues for mitochondrial assays. Although it is possible to make cross-sectional comparisons of animals, these do not reveal individual variations in mitochondrial changes. We and others have partially solved this issue by measuring mitochondrial metabolic traits from biopsy^19^ or blood sampling^40,41^, allowing repeated measurements. However, whether measurements in skeletal muscle or blood cells are representative of mitochondrial metabolic traits in other tissues is unclear, as mitochondrial metabolic traits can differ among tissues^22, 42^ but see^43^. Combining longitudinal and comprehensive analyses across tissues of mitochondrial metabolic traits will be more effective in increasing the understanding of among- and within-individual variations in whole-organism performance^44^.

## Supplementary Information

Supplementary Information 1.

Supplementary Information 2.

## Data Availability

The dataset supporting this article is made available in the Supplementary Material.
