# Supplementary material for: The relationships between growth rate and mitochondrial metabolism varies over time
Source: Sci Rep. 2022 Sep 27;12:16066. doi: 10.1038/s41598-022-20428-9 (PMC9515119; doi:10.1038/s41598-022-20428-9)
Supplement: Supplementary file 2 — Supplementary Information 2. [file 41598_2022_20428_MOESM2_ESM.docx]

**ELECTRONIC SUPPLEMENTARY MATERIALS**

**Effect of biopsy on growth and immune system of fish**

The purpose of this experiment was to determine the effect of a biopsy on the growth and the immune system (Fig. S1) of juvenile European sea bass. Ninety fish were maintained in two replicate tanks, half of the fish (n=45) received a biopsy of red muscle and the other half were used as control. Each fish was anaesthetized, weighed (55.3 ± 1.5g), biopsied or not, placed in a recovery tank before returning to their original tank. The procedure for the biopsy is similar as in the main doc. After 3, 14 and 28 days post-biopsy, fifteen fish from both groups were sacrificed to measure and compare the levels of immune markers in blood, plasma and kidney. The overall immune system response was monitored by measuring the red and white blood cells counts, using a Thoma cell hemocytometer with whole blood drawn from the caudal vein into lithium heparinized vacutainer tubes and diluted to 1/200 in a Giemsa solution (Kekic et al, 1982). The possible biopsy-related inflammation was monitored by measuring the expression levels of interleukin-1 and tumour necrosis factor in the kidney. Total RNA was isolated using an RNeasy Plus kit (Qiagen) from 20 mg of each sampled kidney, and all samples were treated with the RTS DNAse Kit (Mobio) to prevent DNA contamination. [A reverse transcription](https://www.sciencedirect.com/topics/agricultural-and-biological-sciences/reverse-transcription) (RT) was carried out using 1 μg total RNA with a RevertAid H minus First Strand cDNA Synthesis Kit (Thermo Fisher Scientific) and an iCycler System (Bio-Rad). Primers and the characteristics of the quantitative real-time PCR (qPCR) reactions performed in the CFX96 real-time PCR System (Bio-Rad) are available in (Dupuy et al. 2019). The possible biopsy-related infection was monitored by measuring the alternative pathway of complement activity (ACH50) and the concentration of lysozyme in plasma, using a hemolytic assay with rabbit red blood cells (Yano et al. 1992) and a turbidimetric assay with a bacterial suspension of Micrococcus lysodeikticus (Grinde et al. 1988), respectively. ANOVA were performed to compare the level of these immune markers between the biopsied and the control fish across time. No significant difference was observed in the tested markers at 3, 14 or 28 days post-biopsy between the biopsied and the control fish (Fig. S1,B).

**Supplementary references:**

Dupuy, C., Cabon, J., Louboutin, L., Le Floch, S., Morin, T. and Danion, M. Cellular, humoral and molecular responses in rainbow trout (Oncorhynchus mykiss) exposed to a herbicide and subsequently infected with infectious hematopoietic necrosis virus. *Aquat. Toxicol.* **215**, 105282 (2019).

Grinde, B., Lie, Ø., Poppe, T. and Salte R. Species and individual variation in lysozyme activity in fish of interest in aquaculture. *Aquac.* **68**, 299-304 (1988).

Kekic H, Ivanc A. A new direct method for counting fish blood cells. *Ichtyologia.* **14**, 55–8, (1982).

Yano T. Assays of heamolytic complement activity. In: Techniques in Fish immunology (ed. Stolen J.S., Fletcher D.P., Anderson S.H., Kaatari S.L., Rowley A.F. USA: SOS Publications, (1992).

**Figure S1**: Effect of biopsy of the red muscle on the growth and the immune system of juvenile European sea bass – A supplementary group of ninety fish was used to determine the effect of the biopsy of red muscle on the growth performance and immune system. Biopsied fish (n=45) had the biopsy exactly as described in the main manuscript. Control fish (n=45) had exactly the same procedure as the biopsy group but received no skin incision and no tissue collection. Fish were kept in two replicated tank and individually tag. (A): effect of the biopsy of red muscle on the growth performance - Fish body mass was measured three times: 4 weeks before the procedure, on the day of the procedure and 4 weeks after (n=30, 15 biopsied and 15 control fish). Specific growth rate (SGR) was calculated as in the main manuscript, 4 weeks before the procedure and 4 weeks after. All fish gained mass after the procedure, however this gain in mass was slightly lower in the biopsy group compared with the control group (independent *t* = 1.097, *P* = 0.064). (B): effects of the biopsy of red muscle on the immune system. The level of immune markers were measured and compared in both control and biopsied fish at 3, 14 and 28 days post-biopsy. For each measurement, the blood, plasma and the kidney of 15 biopsied and control fish were collected to measure the red and white blood cells counts (RBCC and WBCC), the alternative pathway of plasma complement activity (ACH50), the concentration of lysozyme in the serum, the levels of expression of interleukine-1 (IL-1) and tumor necrosis factor (TNF) in the kidney. Difference between biopsied and control fish across time were analysed with an ANOVA. Change in letter indicate significant results.

A

B

|  | \|  \| Table S1: Results from linear mixed model analyses of future specific growth rates (% day^-1^) in individual sea bass as a function of their initial mass (g), their red muscle mitochondrial density (COX activity), and their red muscle mitochondrial metabolic traits (OXPHOS respiration, ATP production, LEAK respiration). ATP production was determined in pmol ATP s^-1^ mg^-1^ red muscle; COX activity, OXPHOS and LEAK respiration were determined in O_2_  s^-1^ mg^-1^ red muscle. Tank was included as a random effect to control for the order in which fish were processed. Bold denotes significant results. \| \| --- \| --- \| | | | | | | | | | | | |
| --- | --- | --- | --- | --- | --- | --- | --- | --- | --- | --- | --- | --- | --- | --- |
| Future Specific Growth Rates | | Source of variation |  | Parameter estimate ± se | d.f |  | t-value |  | p-value | R² |  | IR² |
| 4 weeks | |  |  |  |  |  |  |  |  | 0.17 |  |  |
|  | | Intercept |  | 2.82×10^-1^ ± 1.07×10^-1^ | 34.00 |  | 2.64 |  | 0.012 * |  |  | 0.02 |
|  | | Initial body mass |  | 9.12×10^-4^ ± 5.75×10^-4^ | 34.00 |  | 1.58 |  | 0.122 |  |  | 0.02 |
|  | | COX activity |  | 7.89×10^-4^ ± 1.44×10^-3^ | 34.00 |  | 0.55 |  | 0.588 |  | < | 0.00 |
|  | | OXPHOS respiration |  | 2.18×10^-4^ ± 1.25×10^-3^ | 34.00 |  | 0.17 |  | 0.863 |  | < | 0.00 |
|  | | ATP production | - | 6.74×10^-5^ ± 2.94×10^-4^ | 34.00 | - | 0.23 |  | 0.820 |  | < | 0.00 |
|  | | ***LEAK respiration*** | ***-*** | ***2.20*×10^-2^ *± 9.90*×10^-3^** | ***34.00*** | ***-*** | ***2.23*** |  | ***0.033 **** |  |  | ***0.10*** |
|  | |  |  |  |  |  |  |  |  |  |  |  |
| 8 weeks | |  |  |  |  |  |  |  |  | 0.08 |  |  |
|  | | Intercept |  | 3.03×10^-1^ ± 7.92×10^-2^ | 34.00 |  | 3.82 | < | 0.001 *** |  |  |  |
|  | | Initial body mass |  | 7.54×10^-4^ ± 4.26×10^-4^ | 34.00 |  | 1.77 |  | 0.085 |  |  | 0.06 |
|  | | COX activity | - | 7.31×10^-4^ ± 1.07×10^-3^ | 34.00 | - | 0.68 |  | 0.498 |  | < | 0.00 |
|  | | OXPHOS respiration | - | 1.18×10^-4^ ± 9.29×10^-4^ | 34.00 | - | 0.13 |  | 0.900 |  | < | 0.00 |
|  | | ATP production | - | 1.36×10^-5^ ± 2.17×10^-4^ | 34.00 | - | 0.06 |  | 0.950 |  | < | 0.00 |
|  | | LEAK respiration |  | 1.39×10^-3^ ± 7.32×10^-3^ | 34.00 |  | 0.19 |  | 0.850 |  | < | 0.00 |
|  | |  |  |  |  |  |  |  |  |  |  |  |
| 12 weeks | |  |  |  |  |  |  |  |  | 0.05 |  |  |
|  | | Intercept |  | 4.39×10^-1^ ± 8.82×10^-2^ | 34.00 |  | 4.98 | < | 0.001 *** |  |  |  |
|  | | Initial body mass |  | 1.11×10^-4^ ± 4.73×10^-4^ | 34.00 |  | 0.23 |  | 0.816 |  | < | 0.00 |
|  | | COX activity | - | 1.50×10^-3^ ± 1.12×10^-3^ | 34.00 | - | 1.26 |  | 0.215 |  |  | 0.04 |
|  | | OXPHOS respiration |  | 9.23×10^-5^ ± 1.03×10^-3^ | 34.00 |  | 0.09 |  | 0.929 |  | < | 0.00 |
|  | | ATP production | - | 3.29×10^-5^ ± 2.42×10^-4^ | 34.00 | - | 0.14 |  | 0.893 |  |  | 0.01 |
|  | | LEAK respiration |  | 2.93×10^-3^ ± 8.15×10^-3^ | 34.00 |  | 0.36 |  | 0.722 |  | < | 0.00 |

|  |  | Table S2: Results from linear mixed model analyses of past specific growth rates (% day^-1^) in individual sea bass as a function of their initial mass (g), their red muscle mitochondrial density (COX activity), and their red muscle mitochondrial metabolic traits (OXPHOS respiration, ATP production, LEAK respiration). ATP production was determined in pmol ATP s^-1^ mg^-1^ red muscle; COX activity, OXPHOS and LEAK respiration were determined in O_2_  s^-1^ mg^-1^ red muscle. Tank was included as a random effect to control for the order in which fish were processed. Bold and asterisk indicate significant results. | | | | | | | | | | | |
| --- | --- | --- | --- | --- | --- | --- | --- | --- | --- | --- | --- | --- | --- |
| Past Specific Growth Rates | | | Source of variation |  | Parameter estimate ± se | d.f |  | t-value |  | p-value | R² |  | IR² |
| 20 weeks | | |  |  |  |  |  |  |  |  | 0.29 |  |  |
|  | | | Intercept |  | 6.57×10^-1^ ± 1.10×10^-1^ | 32.42 |  | 5.97 | < | 0.001 *** |  |  |  |
|  | | | Initial body mass |  | 1.98×10^-3^ ± 1.07×10^-3^ | 24.97 |  | 1.84 |  | 0.077 |  | < | 0.00 |
|  | | | ***COX activity*** | ***-*** | ***6.54*×10^-3^ *± 1.75*×10^-3^** | ***33.51*** | ***-*** | ***3.73*** | ***<*** | ***0.001 ****** |  |  | ***0.12*** |
|  | | | OXPHOS respiration |  | 1.54×10^-3^ ± 1.32×10^-3^ | 32.68 |  | 1.17 |  | 0.250 |  |  | 0.02 |
|  | | | ATP production |  | 3.09×10^-4^ ± 2.81×10^-4^ | 25.08 |  | 1.10 |  | 0.281 |  |  | 0.02 |
|  | | | LEAK respiration |  | 1.03×10^-2^ ± 1.02×10^-2^ | 30.98 |  | 1.02 |  | 0.318 |  | < | 0.00 |
|  | | |  |  |  |  |  |  |  |  |  |  |  |
| 16 weeks | | |  |  |  |  |  |  |  |  | 0.32 |  |  |
|  | | | Intercept |  | 4.70×10^-1^ ± 8.48×10^-2^ | 32.27 |  | 5.55 | < | 0.001 *** |  |  |  |
|  | | | Initial body mass |  | 2.80×10^-3^ ± 7.46×10^-4^ | 24.85 |  | 3.76 | < | 0.001 *** |  |  | 0.09 |
|  | | | ***COX activity*** | ***-*** | ***4.49*×10^-3^ *± 1.30*×10^-3^** | ***33.82*** | ***-*** | ***3.46*** |  | ***0.001 ***** |  |  | ***0.05*** |
|  | | | OXPHOS respiration |  | 1.36×10^-3^ ± 1.02×10^-3^ | 32.95 |  | 1.34 |  | 0.191 |  |  | 0.02 |
|  | | | ATP production |  | 1.01×10^-4^ ± 2.17×10^-4^ | 25.50 |  | 0.47 |  | 0.644 |  |  | 0.01 |
|  | | | LEAK respiration |  | 8.72×10^-3^ ± 7.83×10^-3^ | 30.87 |  | 1.11 |  | 0.274 |  | < | 0.00 |
|  | | |  |  |  |  |  |  |  |  |  |  |  |
| 11 weeks | | |  |  |  |  |  |  |  |  | 0.28 |  |  |
|  | | | Intercept |  | 5.50×10^-1^ ± 8.46×10^-2^ | 30.93 |  | 6.49 | < | 0.001 *** |  |  |  |
|  | | | Initial body mass |  | 1.49×10^-3^ ± 5.79×10^-4^ | 25.24 |  | 2.57 |  | 0.017 * |  |  | 0.02 |
|  | | | ***COX activity*** | ***-*** | ***5.22×10^-3^ ± 1.31×10^-3^*** | ***31.96*** | ***-*** | ***3.98*** | ***<*** | ***0.001 ****** |  |  | ***0.10*** |
|  | | | OXPHOS respiration |  | 1.18×10^-3^ ± 1.02×10^-3^ | 29.98 |  | 1.16 |  | 0.256 |  |  | 0.02 |
|  | | | ATP production |  | 3.25×10^-4^ ± 2.08×10^-4^ | 25.22 |  | 1.56 |  | 0.131 |  |  | 0.03 |
|  | | | LEAK respiration |  | 6.76×10^-3^ ± 7.69×10^-3^ | 28.08 |  | 0.88 |  | 0.387 |  |  | 0.01 |
|  | | |  |  |  |  |  |  |  |  |  |  |  |
| 7 weeks | | |  |  |  |  |  |  |  |  | 0.22 |  |  |
|  | | | Intercept |  | 4.91×10^-1^ ± 7.79×10^-2^ | 31.20 |  | 6.29 | < | 0.001 *** |  |  |  |
|  | | | Initial body mass |  | 1.40×10^-3^ ± 4.51×10^-4^ | 26.35 |  | 3.11 |  | 0.004 ** |  |  | 0.04 |
|  | | | ***COX activity*** | ***-*** | ***4.39*×10^-3^ *± 1.19*×10^-3^** | ***31.07*** | ***-*** | ***3.68*** | ***<*** | ***0.001 ****** |  |  | ***0.09*** |
|  | | | OXPHOS respiration |  | 2.24×10^-4^ ± 9.35×10^-4^ | 29.65 |  | 0.24 |  | 0.812 |  | < | 0.00 |
|  | | | ATP production |  | 2.46×10^-4^ ± 1.89×10^-4^ | 25.93 |  | 1.30 |  | 0.204 |  | < | 0.00 |
|  | | | LEAK respiration |  | 9.17×10^-3^ ± 7.01×10^-3^ | 28.09 |  | 1.31 |  | 0.201 |  | < | 0.00 |
